# Water insecurity among seasonal agriculture workers: perspectives from Spanish professionals

**DOI:** 10.1186/s12939-024-02112-8

**Published:** 2024-02-16

**Authors:** Luis Alejandro Rodríguez-Guerrero, Iratxe Pérez-Urdiales, Astrid Escrig-Piñol, María del Mar Jiménez-Lasserrotte, María del Mar Pastor-Bravo, José Tomás Mateos, Erica Briones-Vozmediano

**Affiliations:** 1 Health Care Research Group (GRECS), Biomedical Research Institute (IRB) of Lleida, Dr. Pifarré Foundation, Lleida, Spain; 2 Department of Nursing, University of the Basque Country, Member of the Biocruces Bizkaia Health Research Institute, Bizkaia, Spain; 3 ESIMar (Mar Nursing School), Universitat Pompeu Fabra-affiliated, Parc de Salut Mar, Barcelona, Spain; 4 SDHEd (Social Determinants and Health Education Research Group), IMIM (Hospital del Mar Medical Research Institute), Barcelona, Spain; 5 Department of Nursing, Physiotherapy and Medicine, University of Almería, Almería, Spain; 6 Department of Nursing, University of Murcia, Cartagena, Murcia Spain; 7 ENFERAVANZA, Biomedical Research Institute of Murcia (IMIB-Arrixaca), Murcia, Spain; 8 Department of Nursing and Physiotherapy, University of Lleida, Lleida, Spain; 9 Society, Health, Education and Culture Research Group (GESEC), University of Lleida, Lleida, Spain

**Keywords:** Transients and migrants, Agricultural workers’ diseases, Water intoxication, COVID-19, Qualitative research

## Abstract

Migrant seasonal agricultural workers face conditions of material vulnerability such as inadequate housing difficulties prevent access to running water supplies. The purpose of this study is to explore the perceptions of professionals involved in the care and support of seasonal migrant agricultural workers, as it relates to water access and water consumption and their impact on these workers’ health, in a context of COVID-19 pandemic. Methods: A qualitative exploratory and descriptive study was conducted in 2021 as part of a larger research project, based on 63 personal semi-structured interviews with professionals who provided support to seasonal migrant agricultural workers in three Spanish autonomous regions. COREQ checklist was used for reporting. The interviews were recorded, transcribed, and imported into ATLAS.ti-9 for an inductive thematic analysis. Results: The results have been structured into two main themes: (1) Accessing and obtaining water; and (2) Health problems related to water consumption. Seasonal migrant agricultural workers experience barriers to obtaining safe water for hygiene, cleaning, food preparation and drinking. The implementation of regulations to reduce COVID-19 transmission resulted in improved hygiene levels in the migrants’ quarters, including access to safe drinking water. Conclusion: This study suggests that water insecurity experienced by migrant seasonal agricultural workers in Spain results from their poor living conditions and causes health problems related to a lack of hygiene and the use of unsafe water. Sustainable solutions are needed beyond the pandemic in order to provide migrant workers with adequate living conditions and ensure their water needs are fulfilled.

## Background

Migrant seasonal agricultural workers (MSAWs) face conditions of material vulnerability, marginalization and social exclusion [1]. These factors, especially as they relate to their irregular legal status, inadequate financial conditions and housing difficulties, force them to live in settlements near work sites during crop seasons. Among other challenges, poor living conditions and financial hardship prevent access to running water supplies, which poses a risk to their health [2]. The vulnerable conditions of MSAWs, together with particularly harsh working conditions, including high temperatures and limited access to hydration, can lead to health problems and increased workplace accidents [3].

According to the United Nations, water security is defined as the capacity of a population to safeguard access to adequate quantities of acceptable quality water for sustaining livelihoods and human well-being [4]. In 2010, the United Nations explicitly recognized the human right to sufficient, continuous, safe, acceptable, physically accessible, and affordable water for personal and domestic use. Sustainable Development GoalTarget 6.1 calls for universal and equitable access to safe and affordable drinking water: safely managed drinking water service, drinking water from an improved water source located on-premises, available when needed, and free from fecal and priority chemical contamination [4]. Deficits in service coverage and quality tend to concentrate on low-income groups, vulnerable groups, and rural populations [5]. 

In towns and cities where people live in low-income, informal, or illegal settlements, they usually have less access to improved drinking water sources than other residents [4]. Lack of access to safe and sufficient drinking water and poor infrastructure limit the ability of MSAWs to prepare food and maintain proper personal hygiene, which is a crucial measure to prevent diseases [4, 6].. Diseases related to water use include malnutrition, neglected diseases, diarrhea, and poisoning, among others, caused by microorganisms and chemicals in drinking water [5]. 

In Spain, it is standard practice to hire MSAWs for agricultural seasons [7]. MSAWs face financial and material barriers related to their low wages, high job insecurity and lack of protection to exercise their rights [8]. Other barriers faced by MSAWs include poor knowledge of the local language, laws and bureaucratic procedures, hindering their access to social welfare and healthcare services [9]. In addition, MSAWs are exposed to geographical barriers and lack of physical means to be able to travel and shop for products to cover their water and food needs [10]. 

The COVID-19 pandemic has aggravated the precariousness of vulnerable people, including migrants [11, 12]. In the United States, regulations aimed to reduce the risk of COVID-19 transmission and improve hygienic conditions were met with implementation barriers [13–15], which resulted in a small impact on long-term hygienic conditions of MSAWs [16]. In Spain, MSAWs were considered to be essential workers [17], which resulted in their increased risk of infection and led to situations of sickness presenteeism of MSAWs to avoid losing their jobs [18]. 

To our knowledge, few studies have been conducted in Spain on water insecurity and its impact on the health of MSAW populations [19]. Therefore, the purpose of this study is to explore the perceptions of professionals involved in the care and support of seasonal migrant agricultural workers, as it relates to water access and water consumption and their impact on these workers’ health, in a context of COVID-19 pandemic.

## Materials and methods

### Study design

This descriptive exploratory qualitative study is part of the first stage of a larger project (AGROMISALUD Project) focused on evaluating the influence of job insecurity and social exclusion on the health of migrant men and women working in agriculture [20]. The study design aimed to collect the knowledge and perceptions of different actors involved in the protection system of MSAWs on the barriers to adequate access to safe drinking water. Semi-structured interviews were conducted throughout 2021 in three Spanish regions with different crop seasons, combining seasonality with intensive agriculture. These regions were chosen because they have the highest concentration of MSAWs. Consolidated Criteria for Reporting Qualitative Research (COREQ) criteria were followed [21]. 

### Participants

Participants were recruited purposively in each province by identifying key informants working in reference institutions and by snowball recruiting. The first informants in each region were selected by convenience or after an internet search. Each participant was asked for additional contacts to invite to participate. The eligibility criteria were working in an NGO, social services, health service, governmental institutions or labor union and having experience assisting MSAWs. Three NGOs from Andalucia rejected to participate, and no one withdrawn during the research. A total of 63 participants (33 women, 30 men) were recruited, including professionals (physicians, nurses, psychologists, social workers, cultural mediators, volunteers, and politicians) from healthcare services (*n* = 21), associations/NGOs (*n* = 31), worker unions (*n* = 8) and governmental institutions (*n* = 3) involved in the care and support offered to MSAWs in three Spanish regions: Andalusia (*n* = 25), Murcia (*n* = 15) and Catalonia (*n* = 23) (Table 1).

**Table 1:** Study participants

**Region**	Sex			Sector				
	Female		Male	Health care	Social services	NGOs	Worker unions	Governmental institutions
Andalusia	18		7	9	1	15	0	0
Catalonia	9		14	8	1	10	2	2
Murcia	6		9	2	0	6	6	1
Total participants *N* = **63**	33		30	19	2	31	8	3

Personal semi-structured interviews were performed until data saturation was reached, meaning that no new information emerged. The research team designed a single script containing open-ended questions focused on the study objectives and based on their previous experience. It contained six sections: care and support for MSAWs provided by the service, migratory trajectory, MSAWs living conditions, MSAWs health, and impact of the COVID19. It was piloted in the first interviews to check its understandability. Additionally, new questions could be added in each interview, following the emerging design of the qualitative research. The interviews were performed between January and October 2021. The duration of each interview was between 40 and 100 min, until reaching discourse saturation. The team of interviewers were part of the AGROMISALUD research project [20]. The interviews were performed both on line and in person in the autonomous regions of Andalusia, Murcia and Catalonia. They were recorded in electronic format and then transcribed literally.

### Analysis

The interview transcriptions were imported into the ATLAS.ti web software, online version 9 [22], used as a support to organize the information before conducting an inductive thematic analysis [23]. As a first step, the authors prepared a code tree based on the interview script, to identify deductively different themes in the transcriptions. They inductively assigned open codes to sentences or paragraphs summarizing their content and organized the codes list under code groups according to their similar meaning. These groups were classified under emerging subcategories. Analytical rigor was ensured by implementing a combination of trustworthiness strategies, including heterogeneity of participant profiles, regions of origin and investigator perspectives. The results are supported with significant excerpts of the interviews, which enhanced the credibility.

### Ethical aspects

The study was conducted following all ethical considerations in the Declaration of Helsinki. Before data collection, approval was obtained from the Healthcare Ethics Committee of the Arnau of Vilanova Hospital in Lleida in 2021 (CEIC-2459). Interviewees voluntarily agreed to participate in the study and signed an informed consent form, including a data confidentiality clause. During data collection and transcription, a unique number was allocated to each participant in order to protect their identities. We removed all textual quotations that could make the informants identifiable.

## Results

The results have been structured into two main theme blocks relating to water access and consumption: (1) Accessing and obtaining water; and (2) Health problems related to water consumption.

### Accessing and obtaining water

#### Water as an unmet basic need

The professionals interviewed in our study identified access to water as a basic need for drinking, cooking and ensuring basic hygienic conditions; however, this need was not always fulfilled.

“*And then, also as a basic need, water is so important for that. Access to water is difficult [for them to obtain] […] But water is so important for them. Sometimes it may be very far [for them to travel] to get drinking water. And they also struggle a lot to get water at least for cooking and drinking*”. (I33, social worker, NGO, Andalusia).

“*These people are ready to work in the fields, buy they need some minimum conditions, such as water to drink, water to wash themselves in their lodgings, some basic hygienic conditions*”. (I11, volunteer, NGO, Catalonia).

Furthermore, access to water was identified as a tool used occasionally by governmental institutions to keep MSAWs away from their territories. For example, the interviewees reported that some local governments were shutting down their drinking fountains to stop migrants from moving from the periphery into the towns.

“*Well, in that case, they go to public fountains if they haven’t been shut down, because a lot of times it’s part of their policy to get them out, get them off the street, to close down the fountains. About toilets, they go wherever they can*. (I11, volunteer, NGO, Catalonia).

It was also reported that some local governments were not equipping migrant settlements with basic living conditions and water supplies, in order to avoid a so-called “pull effect”.

“*Lack of sewage, no water network, no electricity, no waste management, absolutely nothing. This is living in absolute vulnerability of rights, because, to me, putting up a barrel or sewers doesn’t mean a ‘pull effect’, like some mayors say where there are camps*”. (I62, social worker, NGO, Andalusia).

#### Access to drinking water and transportation

Interviewees indicated that MSAW access to water depended on the habitability of their housing units.

“*They don’t meet minimum living standards, because, well, very often there’s no water supply there*”. (I46, social worker, NGO, Andalusia).

“*The settlements were unsanitary, they had no electric supply, no water, and were in hazardous areas, forest areas or right in the middle of the woods*”. (I40, lawyer, NGO, Andalusia).

It was reported that MSAWs were housed collectively in places such as warehouses or industrial buildings, in squats or abandoned houses, slums and shanty towns, with no lavatories or running water for cooking and no nearby access to safe drinking water. In these cases, MSAWs needed to draw on sources of unsafe water such as irrigation pools.

“*Just imagine it. I mean: overcrowding, terrible sanitation, no lavatories, no water for cooking. These are terrible living conditions. Ultimately, this is a source of health problems*”. (I52, social worker, city council, Catalonia).

“*The problem with migrant camps is the absence of services, there is no drinking water, they have to go and collect their water from drinking fountains. If they want to take a bath, they have to use an irrigation pool*”. (I4, medical doctor, healthcare, Andalusia).

*“Horizontal settlements: what we mean by that is these huge sort of shanty towns, old country houses that were virtually in ruins, they have no electricity or water supply whatsoever, there is absolutely no sanitation system*”. (I60, social worker, NGO, Andalusia).

Two factors were identified as determinants of access to safe drinking water: distance between the settlement and the city, town or source of drinking water; and permissibility of access.

“*Drinking water basically depends on that, on how close they are to the town. For example, this settlement in Palo has easier access. Those who are in forest areas cross into nearby properties with 25-liter cans to collect water… But anyway, that depends on the area, on how easily they can get in, and on where their camp is. The closest water source is 7 or 8 kilometers away. And that’s if they even let them take any water*”. (I40, lawyer, NGO, Andalusia).

“*As I said, they take water in those barrels and drag them down from that far, to then boil it and all that. Well, that water is not drinkable or at least there is no guarantee that it’s safe for drinking*”. (I24, activist, NGO, Andalusia).

“*The settlements that were installed around the pavilion did have a water source, which did have safe drinking water. So they refilled their water bottles there*”. (I19, mediator, mediation, Catalonia).

For instance, as explained by interviewees, MSAWs needed to bring back the water they collected in buckets, bottles or fuel cans; they brought these back to their lodgings by foot or by bicycle, and less commonly by car.

“*They’ll bring their water back from whichever tap they can find: there’s the one at San Isidro de Campo Hermoso, for example. They take the water from as close as possible and then bring it back down, they’ll bring it back on their bicycles, which is how they get around most commonly, and they use the water cans.*” (I47, administrative employee, healthcare, Andalusia).

“*They have to go many, many kilometers until they find a water source, and they take their water barrels there. It’s as if they were in their original countries in many cases, or even worse. I mean, many of them are from Guinea-Bissau or Senegal or Mali, and they do have running water back in their cities, don’t they? They don’t have to do that back home*”. (I 24, activist, NGO, Andalusia).

Interviewees identified the use of phytosanitary product containers to carry water as a problem; indeed, even though these containers are larger and more resistant, the water collected in them can become contaminated.

“*The thing is that [phytosanitary] containers are sturdier and they can be carried around every day on a bike. The other ones would break after a few days or a month. And the [large water bottles] from the supermarket are smaller. So that’s why they keep using phytosanitary product bottles*”. (I40, lawyer, NGO, Andalusia).

“*Very often they use containers from phytosanitary products to carry their water around, or else the water, I mean… [from] where they drink the water, it’s not safe water*”. (I2, nurse, healthcare, Andalusia).

In addition, the interviewees reported that, in some cases, MSAWs were obtaining their drinking water as donations from the people living in the area, farm owners or NGO mediators. Most commonly, however, MSAWs were required to buy their water in supermarkets, sometimes up to 40 min away from their lodgings.

“*Well, they take their barrels and go into the town… Some people bring them water, food, and sometimes there’s an agreement between the farm owner and the workers, whereby the owner brings them good food where there’s water, but not every day…*”. (I32, mediator, Catalonia).

“*In industrial buildings, there isn’t, because there’s no supply. In the caves, though, in many of the caves they do have drinking water, and sometimes we went and bought water for them too. We brought them water bottles, a lot of water, so they could wash themselves and also drink it, because they have absolutely no water supply at all, none*”. (I73, social worker, NGO, Andalusia).

“*They did have a fountain, but they told me it was only for washing. For drinking water, they said there’s a supermarket… This water isn’t drinking water, it’s only for washing, they use buckets for that. Yes, usually there’s a tap. This water isn’t, though, this isn’t drinking water*”. (I45, mediator, Andalusia).

It was also pointed out that MSAWs resorted to collaborative strategies such as taking turns to travel to nearby towns for water.

“*So they take their turns and so on. I think they have a very interesting management of solidarity going on*”. (I24, journalist, NGO, Andalusia).

“*Many of them also collect water from the pool that’s been opened up there and they also collect [water] from fountains in the nearest towns… But clearly, some camps are much more privileged and do have a fountain nearby, so they can get their water there, but other places don’t…*”. (I27, nurse, NGO, Andalusia).

#### Access to water during the COVID-19 pandemic

Interviewees identified access to water as the most pressing need during the COVID-19 pandemic. For this reason, some local governments facilitated access to safe drinking water in MSAW settlements. For example, in Andalusia provided water tankers and water barrels, in addition to other services such as waste collection and food distribution.

“*The problem with COVID is that they had no access to daily hygiene, not even regularly, as it should have been, and then the situation got more complicated…*”. (I13, social worker, NGO, Andalusia).

“*Yes, this year, with the pandemic, some councils had to provide water to the people in the camps. Some councils have paid for a tanker that went around the camps bring them drinking water. Other councils put up water barrels and refilled them*”. (I6, nurse, healthcare, Andalusia).

“*They got in some of those tank trucks with water, going back and forth to the town hall, so they had drinking water right there in their settlement. They also distributed food from the councils and collected their garbage, they gave them some garbage containers and collected the garbage…*”. (I33, social worker, NGO, Andalusia).

### Health problems related to water consumption

#### Problems resulting from contaminated water

Because water is used by MSAWs for their personal hygiene, washing kitchen utensils and also drinking and cooking, interviewees emphasized that gastrointestinal complaints had become prevalent because of a lack of access to safe drinking water. Illnesses derived from the use of contaminated water, including skin diseases, were reported.

“*Gastrointestinal problems are common, as people working in this sector will know…*”. (I24, activist, NGO, Andalusia).

“*Because we realized there were many gastrointestinal problems caused by drinking that water they were carrying around in those greenhouse cans…*”. (I48, social integration, NGO, Andalusia).

“*Diseases caused by drinking water from pesticide bottles. There’s a lot of that here. We change their pesticide containers for safe containers. The pesticide containers they use to drink water are causing a huge number of diseases; the skin diseases are also related to using water with pesticides*”. (I12, NGO, Murcia).

“*That will affect their health. If they also add some poor quality water because they’re very heavily contaminated. These are groundwaters. This happened again very near Doñana national park, the groundwaters were used up, they were overexploited and then, of course, there’s the super-intensive use of fertilizers, insecticides and all that craziness, I mean, all of those chemicals, synthetic products, which are used in this kind of agriculture*”. (I29, NGO, Andalusia).

In Andalusia, the water collected by MSAWs from contaminated sources was identified as being of inadequate quality and unsuitable for human consumption, as it could contain carcinogenic chemicals. Moreover, the use of containers intended for storing phytosanitary products such as pesticides was reported as the cause of other medical conditions.

“*People drink water that isn’t safe for drinking. They take water for irrigation and store it in containers for phytosanitary products. It’s full of chemicals and totally poisonous. No matter how hard you wash them, you can’t drink that water; you can’t use it for anything, not even to shower or wash dishes….*”. (I60, social worker, NGO, Andalusia).

“*The problem is they take the water and then store it in phytosanitary product containers, which they rinse out. But of course, it doesn’t matter how well they’ll rinse them, there’s always some chemical residues. What we’ve done as an association is, for example, as an organization we did an analysis of that stored water which they were using, and some of that water was found to be unsafe for human consumption, they had a high percentage of nitrites, which are precursors of some cancers*”. (I27, nurse, NGO, Andalusia).

#### Prevention of diseases related to contaminated water

Some of the strategies described for preventing diseases associated with contaminated water included the supply of safe containers to store water, instead of phytosanitary bottles, and health education. For example, one mediator explained that they had organized workshops with MSAWs to instruct them on how to avoid drinking water from irrigation pools and how to avoid using contaminated bottles for water transportation.

“*When we were distributing water, we also talked to the people so they would collaborate. For example, I don’t know if you’ve been to the San Isidro area, in that settlement they found that contaminated cans were being used– containers for chemical products used for growing tomatoes. They use those to carry their water… Now we buy certified containers and we explain the risks of using contaminated cans; attendees can exchange their containers for good ones*”. (I58, social integration, NGO, Andalusia).

## Discussion

In Spain, MSAWs experience water access insecurity, which hampers their hygiene practices, cleaning, food preparation and drinking. The results of this study emphasize that access to water by MSAWs is not guaranteed due to: (1) the characteristics of the housing units where MSAWs are accommodated, which do not normally have a supply of safe drinking water; (2) the location of MSAW housing units and camps, which influences their ability to travel to nearby sources of water; and (3) the availability of establishments where MSAWs can buy their drinking water. Access to water was identified as a basic need associated to consumption, preparation of food and maintenance of personal hygiene. This is consistent with a study conducted in the United States with migrant farmworkers, which showed that a lack of hygiene was due to a lack of water [24]. 

Regarding the sources for obtaining water, it was shown that access to drinking water among MSAWs was conditioned by the possibilities of buying water. Water is mostly bought by MSAWs from convenience stores near the closest and most accessible town; however, water was also obtained from donations and charity. This is in keeping with the findings of a report on the conditions of MSAW camps in the European Union [25]. Moreover, in line with our results, a study have shown that in rural communities disadvantages migrant and nomadic population rely access to safe water by private means [26]. This could explain the prioritizing acquisition of bottler water reported in the interviews.

Drinking and using water stored in contaminated phytosanitary product bottles has been associated with the development of medical conditions such as skin and gastrointestinal diseases in MSAWs. The use by MSAWs of phytosanitary bottles or pesticide containers for domestic purposes, both for transporting and storing water, has been attributed, among other reasons, to a high level of misinformation about the use of contaminated items [27]. In this regard, contamination by pesticides occurs frequently in superficial waters. This is due, among other reasons, to the proximity between rivers and farms, which helps pesticides to leak through, especially during irrigation seasons [28]. Moreover, the consumption of contaminated or unsafe water increases the risk of infection and gastrointestinal problems; [29] a previous study showed that the lack of water increased the risk of exposure to noxious chemicals [30], which could explain the development of skin diseases reported in our results.

Conversely, the use of water deprivation as a discriminatory control strategy, making it impossible for MSAWs to settle and therefore forcing them to move on, could be comparable to ethnic minority control and segregation strategies employed in other countries [31]. 

Based on the information furnished by the participants in our study, the implementation of COVID-19 prevention measures led to improvements in the MSAW housing units and in baseline levels of hygiene in MSAW settlements, as some of these sites were provided with drinking water and waste collection services, which had otherwise been non-existent. These strategies were implemented similarly in Europe and in the United States; however, they do not appear to have been sustained over time [13]. In this regard, a report published in Spain has proposed some improvements which, if implemented, could serve as a basis for extending the support offered during the COVID-19 pandemic to these populations [32]. 

### Limitations

Some limitations of this study should be taken into account when drawing conclusions. Importantly, because interviews were conducted in the immediate aftermath of the COVID-19 pandemic, some of the observations about improved access to water and distribution of aid may not have been sustained over time. For this reason, we cannot assume that the improvements mentioned by interviewees are free from temporal bias. In this sense, it would be necessary to undertake a second round of interviews to describe the strategies adopted for access to drinking water after the COVID-19 pandemic, in order to establish a comparison. The second limitation refers to the outsider perspective of the professionals interviewed, which should be complemented by the perspectives of MSAWs as the real protagonists of these events. In addition, the experiences of other MSAWs in other Spanish regions could also be explored.

Future studies could also delve into gender-related differences and water needs of MSAWs, specifically in the functioning, behaviors and dynamics in settlements housing women, families with boys and girls, and single men. Regarding these differences, a number of facts remain unknown, such as the proportion of unpaid work taken on by women for collection of water, or the criteria followed by women to decide where to store water and how to distribute it for personal hygiene, cleaning their living quarters, washing their clothes or preparing food, among many other care tasks that are usually imposed on women [33]. 

Despite its limitations in terms of the use of descriptive and cross-sectional data, the originality of our results lies in the compilation of information from different professionals involved in the care of MSAWs, who described and identified multiple types of inequalities, behavioral and material risk factors, barriers, and strategies of MSAWs to access to water in the Spanish context, and how these factors could impact the health of this vulnerable and understudied population. Although some previous reports on the conditions of MSAWs in Spain are cited in this study, we focused on exploring water insecurity in MSAWs. Furthermore, as far as we know, ours is one of the first studies to explore how COVID-19 shaped access to water and hygiene-related aid for MSAWs in Spain. In this sense, participants working in public institutions who planned and implemented real-time measures for an unexpected pandemic described that learning and continuously reviewing protocols would improve performance in subsequent agricultural campaigns.

## Conclusions

The conditions of extreme material poverty which are forced on MSAWs, in addition to the full range of structural barriers faced by them, are determinants of their decision-making for obtaining and using water. This study shows that urgent water-related needs come before disease-preventive decisions on water safety; this occasionally leads MSAWs to forego the risk of contamination.

Water insecurity among MSAWs stems from their precarious living conditions and causes health problems related to inadequate hygiene and unsafe water use. The findings of this study contribute to the knowledge about social inequalities experienced by MSAWs regarding their water needs. Because water insecurity in MSAW populations is a complex problem, sustainable solutions must be sought to ensure that these workers have the necessary living conditions to satisfy their basic needs for water.

Improved water supply and sanitation and better management of water resources are essential for protecting the health of MSAWs, whether used for drinking or domestic use. Lack of safe access to water can lead to increased prevalence of different pathologies, including outbreaks of infectious diseases. Public health services should pay special attention to this population group, considering their social and occupational vulnerability. Moreover, the states should provide financial resources and help capacity-building and technology to provide safe, clean, accessible, and affordable drinking water and sanitation for this population.

## Data Availability

Data cannot be shared publicly because of confidential. Data are available from the Biomedical Research Institute (IRB) of Lleida, Dr. Pifarré Foundation for researchers who meet the criteria for access to confidential data.

## Abbreviations

MSAW: Migrant Seasonal Agricultural Workers
